# Multidrug Antibiotic Therapy for a Non-Human Immunodeficiency Virus-Infected Patient With Clarithromycin-Resistant Disseminated Mycobacterium avium Complex Disease

**DOI:** 10.7759/cureus.18967

**Published:** 2021-10-22

**Authors:** Ai Kawamura, Hitoshi Sugawara, Takahiko Fukuchi, Akira Tanaka

**Affiliations:** 1 Department of Comprehensive Medicine, Division of General Medicine, Saitama Medical Center, Jichi Medical University, Saitama, JPN; 2 Department of Pathology, Saitama Medical Center, Jichi Medical University, Saitama, JPN

**Keywords:** multidrug antibiotic therapy, clarithromycin-resistant mac, vertebral osteomyelitis, chronic necrotizing pulmonary aspergillosis, disseminated mac lung disease

## Abstract

The management of macrolide-resistant *Mycobacterium avium* complex (MAC) disease is challenging. It is extremely rare for non-human immunodeficiency virus (HIV)-infected patients to develop disseminated MAC disease. A 73-year-old non-HIV-infected woman was diagnosed with MAC lung disease (MAC-LD) for 20 years and subsequently chronic necrotizing pulmonary aspergillosis for three years. To avoid drug interaction between rifampicin and voriconazole, MAC-LD was treated with clarithromycin (CLR) alone. The results of the bone biopsy and bone marrow culture conducted for back pain were compatible with CLR-resistant MAC vertebral osteomyelitis. The clinical management of CLR-resistant disseminated MAC disease consisting of lung and spinal lesions with no established treatment and a poor prognosis is challenging. In this case, the patient was treated with multidrug antibiotic therapy, including CLR, ethambutol, rifampicin, amikacin, and moxifloxacin. The results show the effectiveness of multidrug antibiotic therapy in treating CLR-resistant disseminated MAC disease.

## Introduction

The incidence of pulmonary non-tuberculous mycobacterial (NTM) disease has increased 2.6 times from 2007 to 2014 [1,2]. In Japan, *Mycobacterium avium* complex lung disease (MAC-LD) has the highest incidence in pulmonary NTM diseases (88.8%) and is reported to be the most common disease worldwide [1,2]. Disseminated MAC disease frequently occurs in acquired immunodeficiency syndrome (AIDS) patients due to a very weak immune system. However, it is extremely rare for non-human immunodeficiency virus (HIV)-infected patients to develop disseminated MAC disease [3].

The recommended standard treatment for MAC-LD is multidrug antibiotic therapy with clarithromycin (CLR), rifampicin (RFP), and ethambutol (EB) [4]. Voriconazole (VRCZ) is the standard treatment for chronic necrotizing pulmonary aspergillosis (CNPA) [5,6]. The concurrent use of RFP and VRCZ is contraindicated because of a drug interaction in which RFP’s induction of CYP3A4 reduces the blood concentration of VRCZ [7]. In addition, several studies have raised concerns that CLR monotherapy causes CLR-resistant MAC (CR-MAC) [4]. Hence, the clinical management of CR-MAC disease is challenging.

Here, we report the case of a non-HIV-infected patient with CLR-resistant disseminated MAC disease consisting of lung and spinal lesions that developed during treatment for MAC-LD with CLR alone and was complicated by VRCZ-treated CNPA.

This case report was previously presented at the Japan Primary Care Association’s Kanto Ko-Shin-Etsu Block Regional Meeting on November 19, 2017.

## Case presentation

A 73-year-old woman was diagnosed with MAC-LD 20 years ago, but she had repeated interruptions in treatment due to moving and consulting multiple clinicians. During a consult three years ago, MAC was detected twice in sputum culture. Based on the findings of chest computed tomography (CT), she was diagnosed with active MAC-LD. Although *Aspergillus* was not detected in the sputum culture, CNPA was diagnosed based on the chest CT findings and positive serum *Aspergillus* antigen test. Because the concurrent use of RFP and VRCZ was contraindicated, MAC-LD was treated with CLR 600 mg/day, while CNPA was treated with VRCZ 200 mg/day.

Because her lumbago worsened with difficulty in moving after three months, she was admitted to the previous hospital. At this time, a CT scan showed destructive vertebral bulging into the spinal canal at the thoracic vertebra 12 (T12)-lumbar vertebra 1 (L1) level. Metastatic bone lesions or vertebral osteomyelitis were tentatively suggested as the possible causes of lumbago. The results of gastroscopy, colonoscopy, and thoracic and abdominal pelvic contrast-enhanced CT scans were unremarkable for a definitive diagnosis. She was then transferred to our hospital for further examination and treatment.

Upon admission, she was 154 cm tall, her body weight was 37 kg, and her body mass index (BMI) was 15.6 kg/m^2^. Her Glasgow Coma Scale (GCS) score was E4V5M6. In addition, her body temperature was 36.7°C, blood pressure was 110/75 mmHg, pulse rate was regular at 80 beats per minute, respiratory rate was 24 breaths per minute, and SpO_2_ was 98% under nasal cannula at 2 L/minute oxygen.

**Figure 1:**
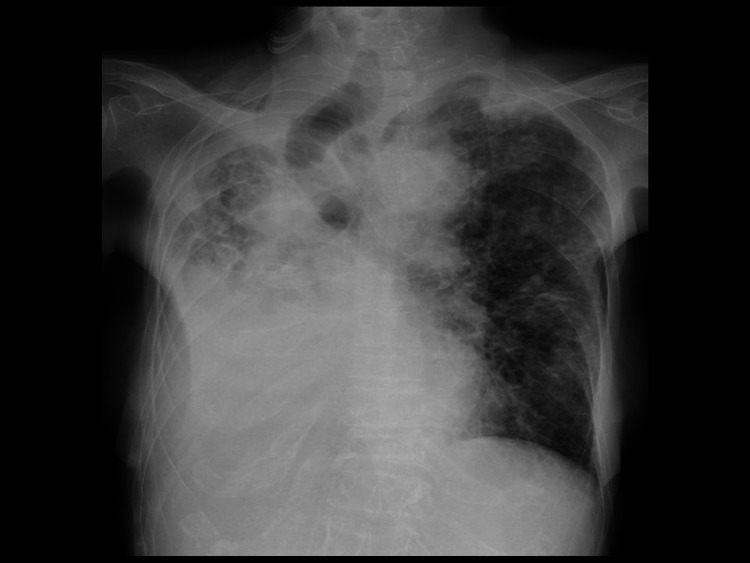
Chest X-ray on admission. Right-sided mediastinal shift and tracheal deviation are observed. The right lung shows atelectasis and infiltrative shadows. The left lung shows ground-glass opacity.

Physical examination revealed pale conjunctiva, no palpable cervical lymph nodes, and coarse crackles in the dorsal bilateral lower lung fields. She had knocking pain in the T12-L1 lesions. Neurological examination showed sensory depression on the dorsal bilateral thigh. The deep tendon reflexes were normal. The laboratory data are presented in Table 1. The chest radiography showed near-total right lung atelectasis and ground-glass opacity in the left lung (Figure 1).

**Table 1:** Laboratory data upon admission. WBC: white blood cells; MCV: mean corpuscular volume; AST: aspartate aminotransferase; ALT: alanine aminotransferase; LDH: lactate dehydrogenase; ALP: alkaline phosphatase; CRP: C-reactive protein; IFN-γ: interferon-gamma; HIV: human immunodeficiency virus; PCR: polymerase chain reaction

Blood cell count			Infectious test		
WBC	8.64	×10³/μL	β-D glucan	6.4	pg/mL
Band	3.0	%	*Aspergillus* antigen	Negative	
Segment	77.0	%	*M. tuberculosis* IFN-γ	Negative	
Lymphocyte	8.0	%	HIV antibody	Negative	
Hemoglobin	8.0	g/dL	*M. avium* PCR	Positive	
Hematocrit	26.6	%	*M. tuberculosis* PCR	Negative	
MCV	78.0	fL			
Platelets	39.1	×10⁴/μL	Culture tests		
			Sputum culture	M. avium	
Biochemistry			Bone marrow culture	M. avium	
Total protein	7.7	g/dL			
Albumin	2.5	g/dL	Drug-susceptibility tests for *M. avium* detected		
Total bilirubin	0.15	mg/dL	Clarithromycin	>32	µg/mL
AST	28	U/L	Ethambutol	16	µg/mL
ALT	16	U/L	Amikacin	8	µg/mL
LDH	177	U/L	Rifampicin	0.5	µg/mL
ALP	366	U/L			

**Figure 2:**
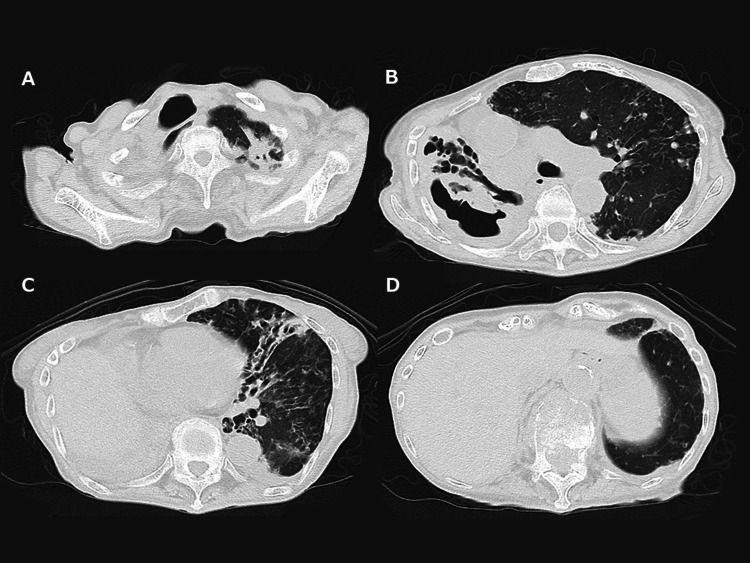
Chest computed tomography scan on admission. A-D: The right lung shows findings compatible with chronic necrotizing pulmonary aspergillosis, such as bronchiectasis, cavities, and infiltrative shadows. The left lung shows findings compatible with pulmonary *Mycobacterium avium* complex disease, such as multiple bronchiectasis, granular shadows, nodular shadows, and cavities.

The contrast-enhanced whole-body CT scan showed findings compatible with CNPA, such as bronchiectasis, cavities, and infiltrative shadows in the right lung, and multiple bronchiectases, granular shadows, nodular shadows, and cavities in the left lung for MAC-LD (Figure 2).

**Figure 3:**
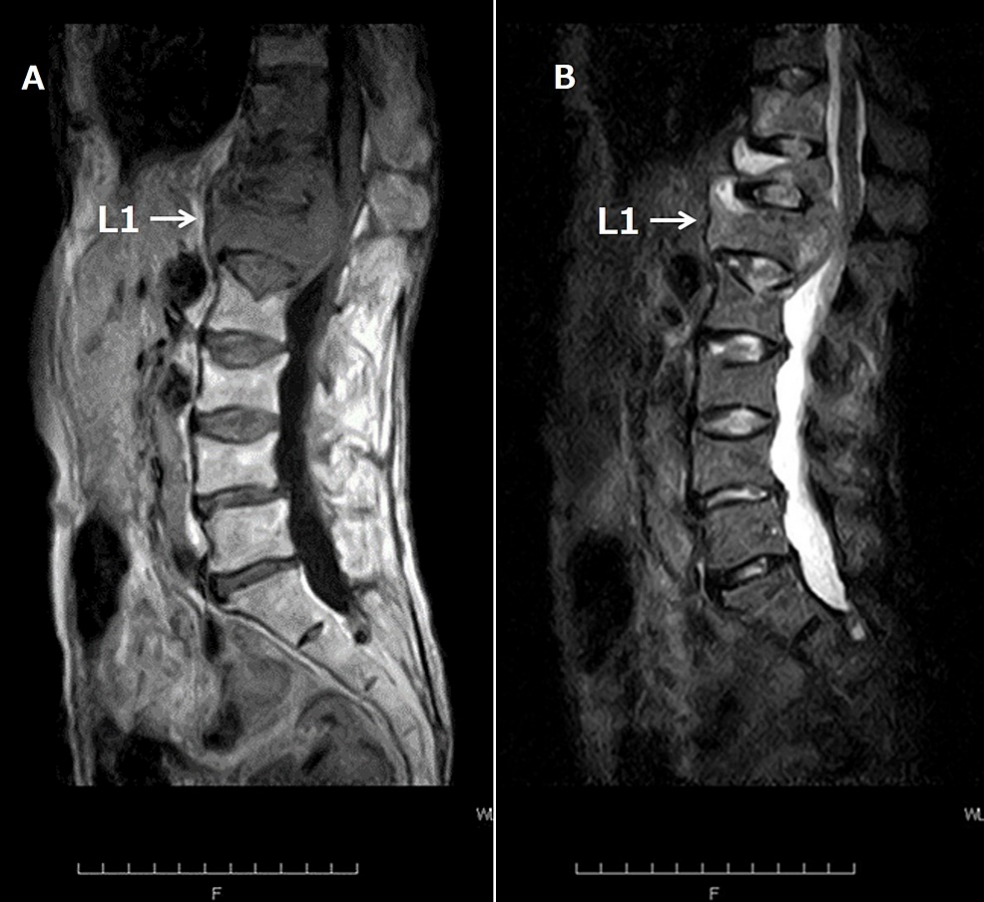
Lumbar magnetic resonance sagittal imaging T1-weighted image (A) and short tau inversion recovery image (B) on admission. (A) T12-L1 has low-intensity lesions. The bone lesions are strongly compressing the dural sac posteriorly and narrowing the spinal canal. (B) T12-L1 has high-intensity lesions. The bone lesions are strongly compressing the dural sac posteriorly and narrowing the spinal canal. T12: Thoracic vertebra 12; L1: lumbar vertebra 1

However, the CT scan did not display any concerning lesions for malignancy or enlarged lymph nodes. The magnetic resonance imaging (MRI) of the spine revealed that T12-L1 had a low-intensity lesion on T1-weighted images (Figure 3A) and a high-intensity area on short tau inversion recovery (STIR) image (Figure 3B). The bone lesion was strongly compressing the dural sac posteriorly and narrowing the spinal canal at T12-L1.

**Figure 4:**
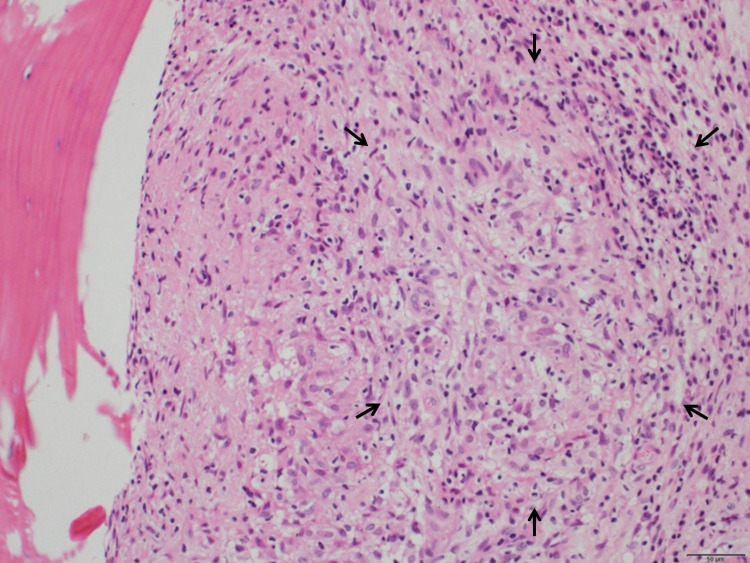
Bone biopsy hematoxylin and eosin stain at 400-fold magnification. Caseous necrosis and a collection of epithelial cells at the margins are observed (arrows), consistent with acid-fast bacterial osteomyelitis. Acid-fast bacterial staining does not reveal any bacteria.

On hospital day (HD) 19, an open bone biopsy was performed at the first lumbar vertebra. Although the acid-fast bacterial staining was negative, the biopsy result indicated acid-fast bacterial vertebral osteomyelitis with caseous necrosis and a collection of epithelial cells at the margins (Figure 4).

The results of the sputum and bone marrow cultures are shown in Table 1.

**Figure 5:**
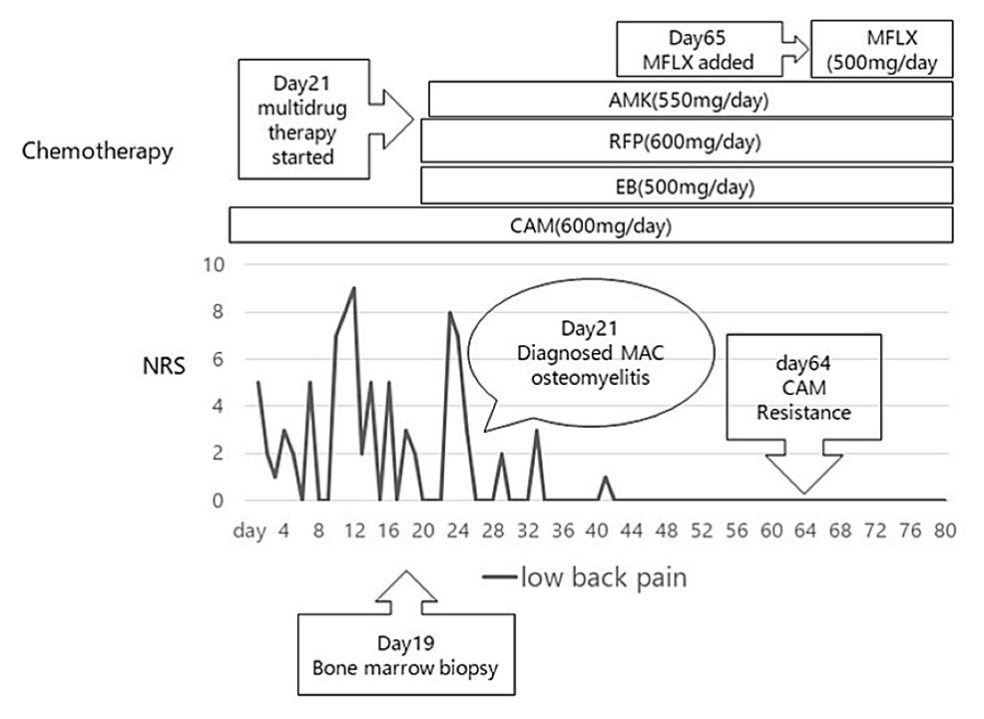
Clinical course after admission. MFLX: moxifloxacin; AMK: amikacin; RFP: rifampicin; EB: ethambutol; CLR: clarithromycin; MAC: *Mycobacterium avium* complex; NRS: numeric rating scale of lumbago

The Ziel-Neelsen staining of the sputum was positive, and the sputum test was negative for *M. tuberculosis* polymerase chain reaction (PCR) and positive for MAC-PCR. *M. avium* was detected in both sputum and bone marrow cultures. Finally, the spinal bone lesions were diagnosed as MAC vertebral osteomyelitis, which was disseminated because of the new bone lesions that appeared despite treatment with CLR 600 mg/day and the treatment for MAC-LD. Because the β-D glucan was within the normal range, with a negative result for serum *Aspergillus* antigen and sputum culture for fungi, we estimated that her CNPA subsided stably, and hence, VRCZ was discontinued. We decided to start treatment with CLR 600 mg/day (16.2 mg/kg), EB 500 mg/day, RFP 600 mg/day, and amikacin (AMK) 550 mg (thrice a week) as multidrug antibiotic therapy for disseminated MAC disease on HD 21. On HD 54, the Ziel-Neelsen stain of the sputum was negative. The minimum inhibitory concentration (MIC) of CLR for *M. avium* isolates was found to be greater than 32 µg/mL on HD 64, indicating CR-MAC disease. Regarding fluoroquinolone antibiotics for MAC, moxifloxacin (MFLX) and sitafloxacin have been reported to be more effective than other fluoroquinolones [8]. On HD 65, 400 mg/day of MFLX was added to the treatment regime. Because two months of treatment reduced her lumbago, she was able to walk with a corset. No adverse drug reactions, such as visual impairment, hearing impairment, or renal dysfunction, were observed. On HD 81, she was transferred to the previous hospital for the continuation of treatment and rehabilitation. She was scheduled to receive at least six months of antimicrobial therapy, along with careful monitoring for clinical signs of soft tissue extension, paraspinal abscess, and cord compression. She needed a nasal cannula of 1.5 L/minute of oxygen. Figure 5 shows the complete clinical course after admission.

## Discussion

This report indicates that the multidrug antibiotic therapy comprising CLR, RFP, EB, AMK, and MFLX, which has not yet been established, led to the remission of CLR-resistant disseminated MAC disease that consisted of lung and spinal lesions. Previously, the patient had CNPA complicated by MAC-LD with repeated self-interrupted treatment. When MAC-LD is treated with first-line regimens including RFP for cases complicated with VRCZ-treated CNPA, the drug interaction between RFP and VRCZ lowers the blood concentration of VRCZ by RFP’s induction of CYP3A4 [7]. EB was not used by the previous doctor. Therefore, MAC-LD was constrained to CLR monotherapy. CR-MAC disease was thought to have developed during repeated interruption of CLR monotherapy due to poor patient adherence. Because of negative sputum culture and *Aspergillus* antigen, inactive CNPA, and normal β-D glucan level, VRCZ was discontinued, and CLR-resistant disseminated MAC disease was treated with multidrug antibiotic therapy. In addition, it was decided to continue CLR treatment based on the reports concerning the improvement of CLR-resistant cases with continued treatment [9]. Regarding fluoroquinolone antibiotics for MAC, MFLX and sitafloxacin were reported to be more effective than other fluoroquinolones [8], and hence, MFLX was added to the treatment regimen.

CLR resistance is assessed by drug susceptibility testing when the MIC of CLR is ≥32 µg/mL [4,10]. The susceptibility results are presented in Table 1. CLR resistance has been attributed to CLR monotherapy, CLR + fluoroquinolone antimicrobials, and the non-use of EB [11]. It has been reported that when MAC-LD is treated with CLR alone, it tends to become resistant within two to five months [4,11]. The treatment with three antibiotics, CLR + EB + RFP, is more effective and less likely to lead to resistance [11]. However, it has been reported that MAC becomes resistant to CLR in 60% of patients, even with these three drugs [12]. EB prevents CLR resistance in AIDS patients (disseminated NTM disease) and in vitro [13], but the frequency of side effects is the highest with the three drugs, with the discontinuation of EB the possible cause of resistance [14]. Although disseminated MAC disease has been reported in AIDS patients, our patient was not HIV positive. The risk factors for disseminated MAC disease in patients without HIV infection include glucocorticoid or immunosuppressive medications, cancer (particularly hematologic malignancy), hematopoietic cell or solid organ transplantation, and severe malnutrition. Our patient had a BMI of 15.6 kg/m^2^ and was malnourished. Although we had no information on whether drug susceptibility testing was not performed 20 years before the patient visited our hospital, if we assume that the patient was not CLR-resistant at the time of the initial visit, CLR resistance may have possibly developed due to the repeated self-interruptions in the treatment, inadequate treatment, CLR monotherapy, and the lack of EB use.

The prognosis of CR-MAC is considered to be poor because macrolides are the key antibiotics for treatment. The prognosis of CR cases has been reported to be 34% at one year and 45% at five years [11]. In MAC-LD, 45% of the patients who failed to receive a combination of surgical resection and long-term treatment with aminoglycosides after the diagnosis of resistance died within two years [11]. The prognosis is poor when surgical resection is not performed in CR cases [4]. Moreover, it is recommended that surgery be considered from the time of diagnosis as MAC-LD may be refractory to multidrug antibiotic therapy [4,15]. For macrolide-resistant MAC disease, it has been reported that sputum antimicrobial activity was mostly negative in cases where CLR was discontinued and high-dose rifabutin and EB were used in combination with surgery and long-term treatment with aminoglycosides [16]. It has also been reported that CLR is effective in CR cases when CLR is continued [9]. In mice infected with CR-MAC that were treated with CLR, CLR reduced the number of bacteria in the liver and spleen, indicating its effectiveness [9]. The American Thoracic Society/European Respiratory Society/European Society of Clinical Microbiology and Infectious Diseases/Infectious Diseases Society of America 2020 guidelines state that injectable AMK or streptomycin (SM) should be added to the initial treatment regimens in patients with cavitary or progressive/severe bronchiectasis or macrolide-resistant MAC-LD [11]. Our patient had a treatment-resistant MAC-LD. In this case, the use of inhaled AMK added to azithromycin, RFP, EB, AMK liposomal inhalation suspension, or intravenous AMK/SM has been reported to improve the sputum-negative rate by the sixth month [17].

It is necessary to pay attention to the adverse effects and drug interactions when complicated patients with MAC start the initial treatment because CLR may cause long QT syndrome [18]; EB may cause optic neuritis [19]; RFP may cause orange urine, tears, saliva, hepatitis, and renal dysfunction [19]; and aminoglycosides may cause hearing loss and renal dysfunction [20]. No adverse effects, such as optic neuritis, hearing loss, or renal dysfunction, were seen in this patient during the multidrug antibiotic therapy.

## Conclusions

Although the treatment of CR-MAC disease has a poor prognosis and no established treatment, the multidrug antibiotic therapy comprising CLR, RFP, EB, AMK, and MFLX was effective in this patient. As drug-drug interactions make drug selection challenging, it is tempting to consider single-agent treatment with CLR, which may lead to resistance. As the treatment of MAC disease is multidrug therapy, physicians must help MAC patients to complete the multidrug antibiotic therapy so that they do not develop antibiotic-resistant MAC. The development of new effective drugs and measures to prevent the progression of resistance to MAC should be attempted in future studies.
